# Hospital and Institutional News

**Published:** 1914-07-25

**Authors:** 


### HOSPITAL AND INSTITUTIONAL NEWS.

### A NOVEL LEGAL DECISION.

DR. LAVERY, the medical officer of the Dundalk Workhouse, recently brought an action against the Dundalk Guardians for fees for attendance on a soldier treated in the infirmary. The Guardians had recovered from the patient a sum of money over and above the average cost of maintenance. Dr. Lavery contended that the excess should be paid to him as the man was obviously not a pauper, and, therefore, not a person whom it was his duty to attend as medical officer. The judge, in giving his decision in favour of Dr. Lavery, said that he felt bound to conclude that the Guardians had received the money on behalf of Dr. Lavery—he did not see for whom else the money could have been received. This decision may lead to similar claims being made in other Unions. Non-pauper cases are not infrequently received into Poor-Law infirmaries and are often charged more than the maintenance rate. It certainly seems equitable that in such cases the medical officer should receive at least some portion of the profit thus made by the Guardians.

### ACTION AGAINST THE BLACKPOOL BOARD OF MANAGEMENT.

THE lengthy dispute between the medical men of Blackpool and the board of management of the Victoria Hospital was again the subject of public interest last week, this time in the Chancery Court of Lancashire, when an application was made to declare void certain elections to the board which were made at the annual meeting last April. The plaintiffs, Dr. R. H. W. Dunderdale and Mr. William John Read, solicitor, both of Blackpool, stated, through counsel, that at the April election three candidates were nominated on behalf of the medical profession, including Mr. Read. The board, continued counsel, looked about for three "friendly" candidates, who were duly elected. The plaintiffs now contended that their election was improper because these three gentlemen were not annual subscribers, and had not subscribed in the period necessary to make them eligible. If these elections were declared void, Mr. Read, a plaintiff, would become entitled to a place on the board. It was also alleged that a proxy given by Dr. Dunderdale, who was entitled to vote as a donor, had been improperly refused by Mr. John Bickerstaffe, the chairman, and that a number of persons were allowed to vote who were not subscribers before or during 1913. In reply to the Vice-Chancellor, plaintiffs' counsel said that even if the plaintiffs succeeded in their claims Mr. Read would still not be elected. The board, he added, had full control of the situation through corporation votes and other means.

### THE BLACKPOOL CASE: JUDGMENT.

IN giving judgment, the Vice-Chancellor said that as the trust deed provided that a donor of not less than ten guineas should be qualified for election to the board of management of the Blackpool Victoria Hospital, and as there was no provision that this sum should be paid as one donation, he held that Mr. Read was qualified. As to whether the three gentlemen who paid subscriptions in March 1914 were entitled to vote and to be elected as annual subscribers, in his opinion a person became an annual subscriber on the day he paid the sum purporting to be an annual subscription, and he had the rights of an annual subscriber for a year from that date. As to the resolution passed in 1895—which limited the qualification to those who paid their subscriptions before December 31—he held it to be *ultra vires*. He therefore thought that the three gentlemen were duly qualified, but expressed no opinion as to whether a person who became a donor or subscriber after an annual meeting was adjourned was entitled to vote at the adjourned meeting. In his opinion a limited company did not come within the clause in the trust deed providing that any subscribing society or institution could authorise a person to vote on its behalf. Even if it did, it could only appoint a proxy under the common seal; and the chairman was wrong, he declared, in admitting proxies which did not comply with that. Both sides had made out some of their contentions, and he should therefore make no order as to costs.

### EMPLOYERS' SUBSCRIPTIONS AND COMPENSATION CASES.

WE publish elsewhere this week a remarkable interview with Mr. Roden Orde, Secretary of the Royal Victoria Infirmary, Newcastle-on-Tyne, on the subject of representation and management. The problems presented to a hospital in a large industrial area where the qualifying subscription for a governorship is extremely low are succinctly stated, and will be of great interest to other parts of England where working men's subscriptions on an organised scale have not been so long in existence as in the North. But there is one passage in Mr. Orde's statement to which we would direct particular attention. It concerns the relation of a voluntary hospital to compensation cases. It is, of course, the aim of the employers to get their accident cases restored to health and work as soon as possible; but for this prompt and expert treatment is required. These two essentials are supplied by the voluntary hospital in so far as funds permit the erection of sufficient accommodation to prevent an enormous waiting-list. A large employer's subscription, then, should be given, from a business point of view, in the light of the amount of compensation money which the hospital saves him by restoring his workmen to health and work. It is the employer's true interest to see that a long waiting-list is abolished, either by new buildings being erected at existing institutions, or by further hospitals being built. But often, so small are employers' subscriptions at the present time, cases are kept waiting for months (during which compensation money is being paid), with the result that it would sometimes pay an employer better to plank down, say, fifteen guineas for prompt treatment at a pay institution rather than to continue compensation money because the local hospital is full. It is poor satisfaction to note that the small subscription of the large employer often defeats its own end by entailing other unnecessary expenditure. The fact remains that enlightened self-interest points to the giving of large annual subscriptions because of the saving which the hospital effects.

### IMPORTANT GIFT TO KING EDWARD VII. HOSPITAL, CARDIFF.

Mr. Lynn Thomas, C.B., F.R.C.S., consulting surgeon to King Edward VII. Hospital, Cardiff, has made an important gift to that institution in the shape of Bedford House, the private hospital which he has conducted for several years. The house, which is valued at £4,000, is an absolute gift to the infirmary, the pathological block of which it adjoins. The grounds are to be partly used for the extension of that block by the addition of lecture rooms for the professors connected with the projected National Medical School. Bedford House itself, by the wish of its donor, is to be devoted to the surgical treatment of paying patients at King Edward VII. Hospital. The house will thus serve the double purpose of providing a portion of the accommodation required at the hospital in connection with the Medical School, and beds for middle-class patients who, while ineligible for free treatment, are unable to afford the high fees of nursing homes. This, we believe, will be the first organised attempt at providing a unit of pay-wards in South Wales at the voluntary hospitals. This gift also signalises Mr. Lynn Thomas's retirement from practice, and will enable him, as president of the Cardiff Medical School, to devote more time to its development, in which he takes the keenest interest.

### GROWTH OF THE PAY-WARD MOVEMENT.

Mr. Thomas's public interests also embrace the Welsh National Memorial Association and the educational work of the University of Wales, while he is now considering a project for forming an association of British surgeons. As house surgeon at the old infirmary in 1883 he has been intimately connected with Cardiff, and, we may recall, during the Boer War he acted as senior surgeon of the Welsh Military Hospital, and for his services received the C.B. Red Cross work has always interested him, and the Voluntary Aid Detachments in Glamorganshire owe much to his help. His investigations into the surgery of goître are well known; but we believe that one of his most important services will prove to be this gift of a unit for paying patients at the hospital. The gap which has hitherto existed in hospital accommodation for the middle classes is being gradually filled, and though the movement is slow, we believe that every fresh extension of pay-wards begets other attempts to supply this need in the voluntary system. A further sum of £10,000 has been presented to the Cardiff Medical School by the same generous and anonymous benefactor from whom so great a sum has already been received. With backing such as this the new Medical School should get a good start, and speedily prove worthy of Wales. Mr. Lynn Thomas, by the way, is also famous as a party to one of the *causes célèbres* of medical jurisprudence.

### THE EAST SUSSEX HOSPITAL FOUNDATION-STONE LAID.

Last autumn, it will be recollected, some heart-burning was aroused in Hastings over certain business arrangements connected with the rebuilding on a new site of the East Sussex Hospital at Hastings (The Hospital, October 25, 1913, p. 86). On July 15 the foundation-stone of the new building was laid with full Masonic ceremonial by the Duke of Richmond, one of the Provincial Grand Masters. About two hundred Masons marched in procession from the Town Hall. The building is to cost £50,000, and is being erected as a memorial to King Edward.

### THE MEDICAL DEPUTATION AND FORCIBLE FEEDING.

Owing to the fact that the deputation from the Forcible Feeding Protest Committee of medical men to Mr. McKenna wished their own reporter, whom they had brought with them, to take notes for publication, their interview with the Home Secretary came to an early close. The latter's view was that as it was proposed to discuss the cases of individual prisoners now under sentence in Holloway he could not consent to the discussion being made public. An official reporter was present, and a complete record of the proceedings would be kept. Mr. McKenna added that he understood that the medical men desired to lay certain views before him, to which he would gladly listen; but he could not publicly discuss with them the cases of individual prisoners now in custody. The medical men—Sir Victor Horsley, Mr. Mansell Moullin, Dr. Wilmot Evans, Dr. Haden Guest, Dr. Moxon, Dr. McLachlan, Dr. James W. McIntosh, and Dr. Schultz—thereupon after a few moments' consideration withdrew. It appears to have been bad tactics, from their own point of view, to withdraw on the above account, though probably Mr. McKenna was the first to be relieved by their decision.

### THE EMPLOYMENT OF CONSUMPTIVES.

Various backwaters of work are now being given to discharged consumptives, and the nature of these occupations varies very considerably. In the provinces certain ex-sanatorium patients recently applied to the local authority for employment in the public parks, and work for more than twenty of the applicants, it is stated, was found; whether it is possible permanently to employ them at this occupation it would be interesting to know. In London, according to Dr. F. J. Allan, Westminster medical officer of health, caretaking has become an occupation for consumptives, either by itself or, more rarely, in addition to other work. Where the houses are unfurnished, he points out that it gives the patients improved housing and causes no risk to subsequent occupiers, but in the case of furnished houses greater care is required.

### THE MEDICAL INSPECTION OF MERCHANT SHIPS.

The value of an expert medical and administrative adviser to every public body of high responsibility we ventured to point out last week when arguing for the appointment of a hospital equerry for the King. What the absence of such an advisory official may mean in the case of a public department is urged by Dr. Hubert Williams, medical officer of the Port of London, in his criticisms of the Board of Trade in reference to its mortality statistics. Now that Mr. John Burns has appointed a Special Committee to investigate the mortality of sailors, and to offer recommendations for alleviating existing evils, Dr. Williams's statement that bad ship construction and antiquated sanitation in men's quarters are always excused by the Board of Trade is already exciting attention. The shipowners of the Bristol Channel have met and passed a resolution describing Dr. Williams's remarks as inconsistent with the facts and as a libel on the shipowners. As these remarks, however, avoided generalities and instanced specific details of construction as habitually faulty, they should prove easy to confute if erroneous; but the main contention on which his argument rests is that "the Board has no skilled sanitary staff and no medical officer to advise it as to ships," with the result that the instructions which it issues to surveyors of ships "are not always carried out." Can it be doubted then that "a skilled sanitary staff" under a medical inspector would improve the mortality rates of merchant sailors?

### HOSPITAL INVESTMENTS.

A point of financial interest was raised at the meeting of the Retford Hospital on the 13th inst. by Mr. E. Wilmhurst, a former treasurer of this hospital. Some years ago at his suggestion the governors appointed legal trustees, and all this hospital's surplus funds were then invested in gilt-edged securities. In the report before the governors that day the accounts showed a sum of £5,712 in Consols. At the date of this purchase Consols were above par, and £6,000 was paid for the £5,712 shown in the accounts. Since the date of this purchase the position of Consols has entirely changed, and the stock which was formerly worth £5,712 was at present valued at £4,421. Thus the caution of the governors of the Retford Hospital in selecting gilt-edged securities has resulted in an absolute loss in capital of no less than £1,579. The point has more than a local interest. It is one which should be pressed continuously upon the mind of the Chancellor of the Exchequer, who is far too prone in all his financial proposals to ignore thrift and to fine the thrifty.

### BOARD'S GIFT TO A HOSPITAL SECRETARY.

At a recent meeting of the governing body of Batley and District Hospital, Yorkshire, it was unanimously decided to give to Mr. Arthur W. Western, secretary of the Batley Hospital, an honorarium on account of "the enormous amount of extra work" which he has undertaken during the past few months. The Batley Hospital is an institution which possesses forty-five beds, and in addition to Mr. Western there is an honorary secretary. It is a striking and graceful tribute that the hospital board should be unanimous in voting this practical recognition of their hospital secretary's work, and illustrates a remark made by a well-known administrator at Newcastle during the recent conference to the effect that he regarded half his work as being paid for and the other half as being voluntarily given by him. He added that he thought this was typical of the relation of salary to services among the paid officers of the hospital world.

### THE WINSLEY SANATORIUM DIFFICULTIES.

About a year ago (The Hospital, July 12, 1913, p. 447) the conversion of the Winsley Sanatorium from a private charity into a municipal tuberculosis hospital was discussed in some detail, and it was pointed out that the Corporation of Bristol were practically usurping the rights of philanthropic people who had raised £21,000 to establish this institution, and had no wish to see it appropriated to relieve the ratepayers of Bristol of their obligations in connection with the Insurance Act. Since then the control of the sanatorium has effectively passed into the hands of the Corporation, and there has been perpetual bickering going on ever since. The medical board are ignored or overridden on technical matters, and at the last annual meeting no report from the Consultative Medical Board was presented. According to the *Bristol Medico-Chirurgical Journal*, this was because none was invited; and previous experience of the mutilation of their reports made the doctors chary of sending in another one unasked. From the same source we learn that the resident medical officer has sent in his resignation, because he found himself hampered by the committee in the control of the patients, and because the number of patients and the lack of trained assistants rendered efficiency impossible. (See also The Hospital, January 24, 1914, p. 443.) "As it is," says the Bristol journal, "the Winsley Sanatorium partakes more of the character of an expensive convalescent home than an up-to-date scientific sanatorium for consumption." The City Fathers of Bristol have assumed the reins of power, and the fact that they did so in a high-handed manner makes their responsibility all the greater for the proper management of the institution. It is "up to them" to disprove the charges now brought against them, or else to reform themselves very thoroughly indeed.

### CINEMATOGRAPHY AT A HOSPITAL OPENING.

A novel point was raised at a recent meeting of the Hull Guardians in regard to an application for permission to take cinematograph pictures of the opening of the new hospital under that authority. One member suggested that the opening of a hospital was a solemn ceremony, and that the cinematograph was out of place there. In fact, there was a great diversity of comment on the proposal, which was eventually sanctioned. Some of the Guardians took the view that the request was of the nature of a compliment, and that it might serve a good purpose in making the hospital more widely known. It is at least difficult to see how it could do any harm.

### A CHEST HOSPITAL TRANSFERRED TO PUBLIC CONTROL.

Following on the passing of the Insurance Act, a meeting of the contributors to the Royal Victoria Hospital for Consumption, Edinburgh, has been held to consider the transference of the hospital, dispensary, and farm colony to the Local Authority for the city, under the Public Health Acts, and to authorise the committee to enter into a provisional agreement. Lord Guthrie reminded the contributors that the first dispensary was opened in 1887, the hospital in 1894, the dispensary in which they were met in 1912, and the farm colony in 1907. The result of the agreement, he said, would be to hand over their three institutions to the Local Authority on certain conditions, and that the Local Authority would undertake the debts on the heritable property and on the administrative side of the work, amounting together to £13,000. On the other hand, there remained with them the whole existing endowments; but while the trustees ceased to hold the properties, the work which Sir Robert Philip had founded would continue on the lines on which it was carried on. The endowments would go not only for the purposes of the institutions, but for after-care and in establishing professorships and lectureships at Scotch universities. Considerable endowments remained for the establishment of a professorship, probably in Edinburgh, to carry forward the investigations. The resolution was agreed to, and the provisional effect of the transference will probably be seen at the beginning of August. The Royal Victoria Hospital will continue as a trust, to be known as the Royal Victoria Hospital Tuberculosis Trust, one of the purposes of which will be to assist consumptive patients throughout Scotland who are not provided for by statutory authority. The public are being appealed to to continue their subscriptions so that the combined treatment and scientific investigations may be carried on over a wider area than before, and, for instance, some provision be made for advanced cases. The latest great development of a scheme which began as the first tuberculosis dispensary in Great Britain will be watched with much interest.

### THE LAST REPORT OF THE LUNACY COMMISSIONERS.

It is mainly a sentimental interest which attaches to the last Report of the Lunacy Commissioners, whose duties since April 1 became merged in those of the Board of Control appointed under the Mental Deficiency Act. The increase in insanity is again noticed, though possibly the more active attempts to control affected persons during recent years may help to make this increase more apparent than real. It is characteristic of the changes of opinion in regard to mental disease that the Lunacy Commissioners' dying breath, so to speak, conveys a plea for the treatment of incipient cases, which is one of the aims of the Act which supersedes them. As our readers recall, we have recently considered the problems arising out of an attempt to receive and treat such cases without the stigma of certification. The Report states that the ratio of the insane to population since 1859 has increased from 18.67 per 10,000 to 37.59. That is the central fact of their record, and forms the text on which they urge that institutions should be considered, not as places of detention, but as mental hospitals, an aspiration with which we have the greatest sympathy.

### MENTAL WARDS IN WORKHOUSES.

Mr. B. T. Hodgson, a Commissioner of the Board of Control, Local Government Board, recently visited the lunacy wards of the Lambeth Workhouse, which have at times been overcrowded, and in the course of his report he raises an important point as to certification. Some of the orders, though signed by a Justice, were not dated, and the name of the asylum in which the patient was to be received had not been filled in. He was given to understand that the date and the name of the institution would be added by the relieving officer when the vacancy had been allotted. He explained to the medical officer, Dr. Baly, that this procedure was quite irregular, and that he had no authority to detain the patients, and, until a valid order was in existence, he should legalise the detention by a fourteen-day order given by himself. The wards were clean and tidy, and he was satisfied that the inmates of them were receiving proper care and attention, but the wards are not suitable for the purpose to which they are put, all kinds of cases having to be mixed up in them, young children with drink and violent cases. He was informed by the medical officer that the wards also are often overcrowded. The beds and bedding were clean, and in good order, new beds having been provided on the female side but none of the beds had under-blankets. The staff consists of two by day and two by night in each ward. The Guardians' board decided, with reference to the question of procedure, to instruct the officers to do their best to comply with legal requirements in these cases. As to the point of the adequacy of the accommodation, the Guardians are already considering steps to increase it.

### THE "PARADE SEASON."

The season of Saturday fêtes and parades is once again in full swing in Leicestershire. As a means of helping the funds of the Leicester Royal Infirmary last year these parades were productive of great results, and as interest increases year by year it looks as if a useful permanent source of income has been found. Besides helping the cause of the hospitals, we are pleased to chronicle at least one instance where parade committees have done useful service in the public weal. At Bagworth, a colliery village, the local committee (most enthusiastic workers all) have actually during the season between one parade and another, by means of concerts and other efforts, raised sufficient money for the building of a bandstand, which they have presented to the parish—an incident showing what a wealth of useful service there is in well-directed effort. We hope later on to give the results of these parades in Leicestershire, but in the meantime we suggest to hospitals seeking a lively source of income to inquire particulars of these Leicestershire parades taken up so enthusiastically by the residents.

### HOSPITAL OFFICERS AT PLAY.

The Hospital Officers' Golf Tournament has brought into notice some keen exponents of the game, for there have been many tough fights in the struggle for the "President's" Challenge Cup, which has now reached the semi-final stage. So fired by enthusiasm have some hospital officers become that at one hospital the "clock golf" in the garden now serves a dual purpose, for it marks the last green of a miniature golf course—miniature, indeed, for the longest hole cannot be more than thirty-five yards. However, a full drive with a captive ball can be taken, and then an ordinary ball is dropped for the approach shot of a yard or two to the green. The "bogey" for the course is a very difficult ten. It certainly needs a little imagination, but it provides good practice for idle moments.

### THE QUESTION OF SPECIAL DEPARTMENTS FOR VENEREAL DISEASES.

At St. Mary's Hospital a proposal is under consideration for creating a special department for venereal diseases, in charge of a specially appointed member of the staff; and a night clinic is one of the features of the scheme that is formulated. To this scheme Dr. Graham Little, the dermatologist to the hospital, objects very strongly in a letter which the editor of the *St. Mary's Hospital Gazette* has published in the June number of that journal. Syphilis and gonorrhœa have, until quite recently, been allotted by tacit convention in London institutions to the care of the general surgeons, though there is really no logical justification for this course. The dermatologists have not infrequently found cases of syphilis in their clinics, and, being every whit as well able to diagnose and treat the disease as a general surgeon, they have commonly done so, and have in consequence come to regard syphilis as falling within the limits of their speciality. In the last few years two new factors have come into play: one, the new discoveries in the diagnosis and treatment of syphilis, and the other the creation of departments at many hospitals for genito-urinary diseases. Added to these have been the universal tendency to specialism, and the work of the Committee now sitting. The result is that general surgeons and dermatologists are seeing much less syphilis than they used to do. Dr. Little's arguments against the new proposal are, therefore, perhaps open to the suspicion of being special pleading; and certainly some of them do not hold water, as is pointed out editorially in the *Gazette*. Still, there is distinct cogency in some of his remarks, particularly when he pleads for very careful consideration without haste of a project which certainly creates precedents and requires examination from every possible point of view.

### THE POWER OF A LINEN LEAGUE.

To enlist the services of ladies in the work of a hospital is an unquestionable aid. Some 300 comprised the gathering in the out-patients' hall at the annual meeting of the Needlework and Linen Guilds' Association, held in connection with King Edward VII.'s Hospital, Cardiff, last week, under the presidency of Mrs. Trevor Tyler, who was supported on the platform by Lieut.-Colonel E. M. Bruce-Vaughan, and by the presidents of the Guilds, Lady Courtis and Mrs. Henry Lewis (Greenmeadow). The reports presented showed that no fewer than 489 garments and 1,300 articles had been given, and that the value of the gifts had increased. It was at this meeting that Lieut.-Colonel Bruce-Vaughan announced the two great gifts we have mentioned above.

### FURTHER BEQUESTS FROM SIR ALFRED JONES

A second scheme of benefits to charities—amounting to £24,350—was presented this week at the Liverpool Chancery Court under the will of the late Sir Alfred Jones. The forty-three charities which will benefit include the Liverpool School of Tropical Medicine, while the Garston Hospital, which received £10,000 under the original scheme, is to be given an additional £1,000. Under the first scheme £227,000 was distributed, and the death of the widowed sister of Sir Alfred Jones now sets free this further sum; and a sum of £50,000 or more is expected to be available for a third scheme of distribution. The institutional bequests comprise £5,000 to the Liverpool School of Tropical Medicine, £2,000 to the Leasowe Sanatorium for Crippled Children, Cheshire, where a block of forty-four beds is to be built in memory of the testator, and £2,000 to the Victoria Central Hospital, Liscard. The sum of £1,000 is also given to the King Edward VII. Welsh National Memorial Association, to the Liverpool Royal Infirmary, to the Carmarthen Infirmary, and to the Birkenhead Borough Hospital. The will is a fine tribute to generosity and public spirit.

### SCOTCH SERVANTS AND HOSPITAL PROBLEMS.

According to the by-laws of the Clackmannan County Hospital, Scotland, domestic servants, qualified for admission, may be admitted at the instance of their employers on payment of from seven shillings to ten shillings a week for maintenance, subject to appeal to the house committee. The passing of the Insurance Act has, however, led certain employers of servants to suppose that if they paid the weekly charges under the Act they would be relieved of their former liability under the common law to support a domestic servant for six weeks during ill-health. The result has been that several employers whose servants have been taken to the hospital during the present year have declined to pay the treasurer's accounts for their maintenance. The managers were of opinion that the by-law should now be repealed, but the hospital secretary, Mr. C. Thomson, strongly asserted that the employers' liability to maintain their domestic servants for six weeks in sickness was unaffected by the Insurance Act. Since Lord Balfour of Burleigh was present at the last quarterly meeting he agreed to take counsel's opinion on the matter, and has written stating that he is satisfied after inquiries that the secretary's view of the matter is correct, This is a feather in the cap of Mr. Thomson, and should have the effect of getting the hospital's charges being promptly met. The situation, however, was clearly open to divergent opinions, and we can understand some thinking that the common law and the Insurance Act were overlapping one another.

### THE NEW WORKHOUSE HOSPITAL AT HULL.

The Lord Mayor of Hull last week opened the new workhouse hospital in the grounds of the Hull Workhouse. It is built behind the workhouse and consists of four storeys, with the administrative block in the centre, from which radiates to the right and left a corridor with main wards containing thirty-one beds on each floor of the centre block. These wards, which are ninety-six feet long, are supplemented by two isolation wards, and the top floor, half of which is open, is reserved for consumptive cases. The total number of beds founded is 235, and the cost, exclusive of the site but including the equipment, is given as £20,000. It is asserted that the £85 per bed, which is the cost represented, makes the institution the cheapest of its class under the Poor-Law. Between the workhouse and the new sick wards gardens have been laid out, and the new building itself is simple and not unattractive. Councillor W. Wheatley has acted as the chairman of the extension committee, with Councillor Watson Boyes as deputy, and the architect is Mr. T. Beecroft Atkinson, Trinity House Lane, Hull. If only all the fine buildings under the Poor-Law were used as genuine hospitals, instead of as, so often, homes for chronic cases, there would be even more cause than there is to welcome up-to-date design in Poor-Law extensions.

### THE VISITATION OF PUBLIC INSTITUTIONS.

The Camberwell Board of Guardians have been endeavouring to persuade the Metropolitan Asylums Board to allow them to send visitors to their various institutions where people from Camberwell are under treatment. They were not desirous of visiting the infectious hospitals. The Board, in reply, point out that if each Board of Guardians made a similar request, and the applications were acceded to, it would mean that twenty-eight different committees would visit most of the institutions during the twelve months, and that the time of the officers at the institutions would be taken up to an extent which would seriously interfere with the performance of their duties. The managers also point out that the Guardians have representatives on the Board who visit the institutions, can interview Camberwell patients and obtain any information which the Guardians might desire.

### A HOSPITAL EQUERRY FOR THE KING.

The suggestion which we put forward in our last issue that a hospital equerry should be attached to the King to bring before his notice, even more fully than is at present possible, the latest points of interest in the institutions which he visits, is much criticised by a medical contemporary. Unfortunately the arguments adduced —that such an appointment could only lead to requests for similar representatives for all and sundry social activities—is followed by an attack on the King Edward and Metropolitan Sunday Funds, which are unjustly accused of unfairness to the smaller institutions. The connection between the preceding argument and the criticisms which succeed it are not close, but because the argument is put forward it may be answered briefly: The voluntary hospitals, ever since the late King when Prince of Wales manifested a keen personal interest in them, stand in a different position to the Throne from that occupied by any of the other social forces mentioned. No other type of institution is so closely connected with our Royal Family, nor does any hold the same place of respect in popular regard. Of what other institutions do members of the Royal Family become active chairmen?—what others possess a fund personally founded by King Edward?—what others, again, are associated with charity to the same extent? The hospitals have a more intimate relation to our Royal Family than any other movement of equally wide appeal, and their personal interest in maintaining the hospital standard may well suffice to justify the proposal that there should be a hospital equerry for the King.

### THIS WEEK'S DRUG MARKET.

Few changes in the values of drugs have taken place, and the market continues quiet. Peppermint does not promise well, and it is expected that the prices for English peppermint oil will be higher. Belladonna is only fair. Henbane has been a poor crop in one district and a good average one in another. On the whole lavender looks fairly promising, but the frosts at the end of May did some damage in at least one growing district. Chamomiles would have been better with more rain. Marshmallow, wormwood, rue, comfrey, and yarrow are satisfactory. The expectation that the price of cocaine would be advanced has been fulfilled, but quotations are still sufficiently low to tempt buyers to replenish their stocks. The higher value of opium is well maintained, as also are the quotations for morphine and codeine. Essence of lemon continues to tend lower in price. Citric and tartaric acid continue in good demand. Menthol is unchanged. Thymol is dearer.

